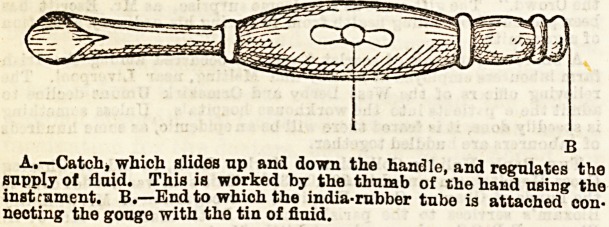# The Discussion on the Treatment of Spinal Abscess at the Nottingham Meeting of the British Medical Association

**Published:** 1892-08-27

**Authors:** 


### The Practitioner's Mirror and Retrospect.

[*The Editor will be glad to receive offers of co-operation and contributions from members of the profession. All letters should be addressed to* The Editor, The Lodge, Porchester Square, London, W.]

### SURGERY.

### THE DISCUSSION ON THE TREATMENT OF SPINAL ABSCESS AT THE NOTTINGHAM MEETING OF THE BRITISH MEDICAL ASSOCIATION.

A very interesting discussion took place at the Nottingham meeting of the British Medical Association, on the treatment of spinal abscesses. In the older days of surgery, many surgeons dreaded to open these collections of pus, and they were allowed to burst, so as not to hurry their evacuation, for it was believed that the longer they remained unevacuated the better for the patient. Many surgeons believed that when once a psoas abscess began to discharge, the patient began "to go down hill" very rapidly. All this has now been changed by the introduction of antiseptic surgery. We are no longer afraid of opening these abscesses, but we are very much afraid of allowing them to discharge spontaneously, and so become septic. It is still rather a doubtful question whether we should cut deeply in order to get at them before they approach the surface, *i.e.*, when they can be felt deeply in the iliac or lumbar regions, but when they present in the usual position on the thigh there is no question that they should be antiseptically evacuated.

We must distinguish between these collections of pus and true abscesses. Indeed, some pathologists would hardly grant them the name of abscess. They are the product of breaking down tubercular tissue, not pus such as we meet with in the acute infective septic abscess swarming with micrococci of an infective nature.

It is not enough, as Mr. Watson Cheyne remarked, merely to evacuate the contents of these spinal purulent collections, we must also get rid of the tubercular granulation tissue lining the abscess sac, and this is best done by flushing and scraping. First, as to the scraping. The most convenient instrument is Barker's flushing gouge, Fig. A. The current of fluid passes through the gouge as it is

B

A.—Catch, which slides up and down the handle, and regulates the supply of fluid. This is worked by the thumb of the hand using the instrument. B.—End to which the india-rubber tube is attached connecting the gouge with the tin of fluid.

at work, and washes away the *debris* as it is scraped off the wall of the cavity. The strength of the current can be regulated by the catch in the handle, which is worked by the same hand that holds the instrument. The instrument can be made of any length or curve so as to get to all parts of the cavity, and it would be a good plan to have several of various lengths and curves at hand. This is the flushing gouge which Mr. Barker uses for his very valuable method of hip excision, which we described in The Hospital early in the year.

So much for the gouge: now we come to the flushing solution. Boiled water, warmed, is an aseptic and non-irritating fluid, and is, therefore, well adapted for the purpose, but it is apt to run short, and so sometimes a 1 in 10,000 solution of corrosive sublimate, which is also aseptic, though not strong enough to be antiseptic or irritating, may be used instead, warmed. It would not be wise to inject cold fluid so freely as would have to be done into so large an abscess sac in the abdomen. The water is used at a temperature between 105 deg. and 110 deg. Fahr. It is supplied from a tin holding about two gallons, suspended so that the fluid may come with some force along an india-rubber tube connecting the tin with the flushing gouge. This must be of some length so as to give free play for the instrument.

After the abscess wall has been thoroughly scraped and flushed out, a drainage tube must be inserted, but only long enough to reach the cavity; we do not want a tube lying in the cavity and acting as a foreign body there.

Now so long as we can keep the cavity aseptic, the chances of recovery are very fair, much greater than they used to be in the days of septic surgery, and a very considerable proportion of cases so treated under Professor Lister and Mr. Watson Cheyne recovered. Once let them get septic, and their chances are at once diminished. Before opening them, the skin must be carefully washed with soap and warm water, perhaps scrubbed with a nail brush. Then it is best to apply to the area to be included in the dressing lint soaked in 1 in 20 carbolic solution for some hours before operation. The instruments are placed in 1 in 40 carbolic solution, and the hands of the operator and his assistant thoroughly scrubbed with soap and water and then well dipped in carbolic solution of the same strength. Recent investigations show the great importance of thorough washing of the hands and nails, perhaps even more than the antiseptic dipping, which should, however, never be neglected. Towels rung out of hot carbolic solution (1 in 40) should be placed around the seat of operation over mackintoshes in case instruments are thrown down for a time. The drainage tube should be kept soaking in a carbolic solution (1 in 20), ready for use. A piece of the "green protective is placed over the wound around the drainage tube, a hole being cut in it to receive the tube; and then a bit of gauze, wet with carbolic lotion, is placed over the tube and wound, and then a large mass of blue sal alembroth wool to absorb the discharge, and finally a Jackonet covering, which being waterproof compels the discharge to soak to the edge of the mass of antiseptic wool before it can get to the air, and will not allow it to run straight through the centre, which would be, of course, the quickest route, too quick to allow enough antiseptic wool to intervene between the point where it came in contact with the septic materials and the wound. Besides the ordinary gauze bandage, it is well to fix the edges of the dressing with a rather narrow elastic bandage, not as strongly elastic as a Martin's bandage. Bandages are sold for this purpose. We believe the elastic outer bandage to be very important. The gauze bandage gets loose and the edge of the dressing is apt to be turned up, and so the discharge is exposed to the air before it has penetrated to the edge of the dressing, or the dressing is apt to slip. These things are prevented by the use of judiciously applied elastic bandages.

The dressing must be examined daily to see if any discharge has soaked through to the edge, and if so a fresh dressing must be applied, the skin around the opening being washed with antiseptic lotion, 1 in 40 carbolic or 1 in 2,000 corrosive sublimate solution; and some surgeons dust on iodoform just around the drainage tube, which must be taken out and washed in the antiseptic fluid. A safety-pin which has been thoroughly soaked in carbolic solution run through the end and fastened prevents it slipping into the abscess cavity. Even if the discharge does not come through, a week is the longest time it can safely be left unchanged. Towards the end of a case it may often fail to appear in this time, though at first daily dressings are generally required.

Some surgeons are not content with scraping and flushing the sac, they inject iodoform as well. At the discussion at Nottingham there seemed a general impression that cases did better with iodoform than without, though the speakers hardly knew why. After the scraping and flushing, a glycerine emulsion of one part of iodoform to ten of glycerine by weight is injected so as to run into all parts of the cavity and coat its walls, and then allowed to run out again. A solution of iodoform in ether is sometimes used, but the distention of the cavity from the expanding ether is not desirable, and in some cases sloughing has resulted.

All this—the scraping, flushing, and iodoform injection—sounds very like a description of Barker's hip excision, and they are all important steps in that procedure; but, alas, the adaptation of the walls of the cavity after thorough removal of the whole disease cannot be applied to the spinal abscess. We cannot approximate the walls by carefully adjusted pressure as we do in the hip, though if we made a lumbar or iliac opening we certainly could bring together the walls of that part which is in the thigh, *i.e.*, if it has extended so far; nor can we in the spine get away the bone disease. But this brings us to an important question. How far can we deal with the bone disease. Sometimes we can get away good-sized sequestra through a lumbar opening, but not often; we might even scrape away carious bone from the front of the bodies if in the lumbar region, and we could (through a lumbar incision) get a finger on to them to guide us, but we cannot do much bone scraping without knowing where we are going, for we have seen the cord in its case of dura mater run uncovered through a cavity between the bodies of the vertebræ, when a carious body has become disintegrated. But the possibility of removing sequestra seems to us greater through a lumbar wound than any other, as we get here nearest to the spine, and we have seen large sequestra removed in this way, though with considerable difficulty. Another advantage of the lumbar over the iliac openingis its dependency. Both lumbar and iliac openings are much better than openings in the thigh, which are liable to contamination from urinary or fœcal excreta. In children we have several times seen cases get septic in this way.

Rest is essential in combination with this treatment of the abscess cavity. Mr. Watson Cheyne keeps his patients in bed, sometimes for many months or a year or two, and does not think they suffer from it in other ways. Some surgeons put them upon plaster jackets and do not keep them in bed. Both plans were advocated at the discussion, but the need for rest to the spine is, of course, universally recognized.

Billroth's method of treating these abscesses by aspirating, then leaving iodoform emulsion in the sac, was not much spoken of, although in Billroth's hands the method seems to have been very successful.

## Figures and Tables

**Figure f1:**